# Death attitudes and associated factors among health professional students in China

**DOI:** 10.3389/fpubh.2023.1174325

**Published:** 2023-05-25

**Authors:** Huiwu Han, Ying Ye, Hongxia Zhuo, Shaohui Liu, Fan Zheng

**Affiliations:** ^1^Teaching and Research Section of Clinical Nursing, Xiangya Hospital of Central South University, Changsha, Hunan, China; ^2^Xiangya School of Nursing, Central South University, Changsha, Hunan, China; ^3^Health Management Department, Xiangya Hospital of Central South University, Changsha, Hunan, China; ^4^National Clinical Research Center for Geriatric Disorders, Xiangya Hospital of Central South University, Changsha, China

**Keywords:** death attitudes, associated factors, health professional students, advance care plan, death-related experiences

## Abstract

**Background:**

China is entering an era of aging population with an increased mortality rate among this category of population. Health professional students' attitudes toward death directly affect their quality of palliative care in their future careers. It is thus important to understand their death attitudes and associated factors to guide future educational and training development.

**Objectives:**

This study aimed to investigate death attitudes and analyze the associated factors among health professional students in China.

**Methods:**

In this cross-sectional study, 1,044 health professional students were recruited from 14 medical colleges and universities. The Chinese version of the Death Attitude Profile-Revised (DAP-R) was used to evaluate their death attitudes. A multiple linear regression model was used to analyze the influencing factors of attitudes toward death.

**Results:**

Health professional students tended to accept death more neutrally. Multivariate analysis showed that their negative death attitudes were associated with age (β = −0.31, *p* < 0.001) and religious belief (β = 2.76, *p* = 0.015), while positive death attitudes were associated with age (β = −0.42, *p* < 0.001), hearing of Advance Care Plan (ACP) (β = 2.21, *p* = 0.001), and attending funeral/memorial services (β = 2.69, *p* = 0.016).

**Conclusion:**

Our study stresses the importance of including death and palliative care education in healthcare courses among health professional students in China. Incorporation of ACP education along with experiences of funeral/memorial services may help promote health professional students' positive attitudes toward death and improve the quality of palliative care in their future careers.

## Introduction

China is entering an era of aging population. According to the data released by China's National Bureau of Statistics at the end of 2022, the natural growth rate of the Chinese population is −0.60‰—the first time China has seen a trend of negative population growth since 1962 (1). Death is a natural process for all human beings. Although it is known that death is inevitable, people's attitudes toward death vary from culture to culture (2). Compared to Western culture, death is still considered a taboo in traditional Chinese culture (2). Ever since the outbreak of the COVID-19 epidemic in 2019, there has been a notable rise in COVID-19-related mortality among the aging population, which shed light on the urgent need for high-quality end-of-life care.

Death attitude refers to people's emotional reactions when facing the near-death and death stimuli of themselves or others. It is a multidimensional concept that reflects a stable and evaluative psychological tendency and can be generally classified into positive and negative death attitudes. According to the Death Attitude Profile—Revised (DAP-R) developed by Wong et al. (3), positive attitudes include neutral acceptance, approach acceptance, and escape acceptance, while negative attitudes include fear of death and death avoidance. Neutral acceptance refers to the view that death is an integral part of life. Approach acceptance is defined as the belief in a happy afterlife after death. Escape acceptance is the consideration that death is a welcome alternative to a life full of pain. Fear of death is marked by the feelings of fear evoked by confronting death. Death avoidance is characterized by the avoidance of thinking or talking about death to reduce the emotional distress caused by death.

The student period of health professionals is the key period of professional personality shaping. Health professional students will inevitably encounter the death of patients in their future careers, and it is thus important to understand their attitudes toward death during the student period (4). Health professional students' attitudes toward death directly affect their values of life and patients' cognition of death and thus influence the quality of end-of-life health services (5). Health professional students' negative attitudes, such as death avoidance and anxiety (6), may make dying patients doubt their self-worth and interfere with their self-transcendence (7). On the contrary, health professional students with positive death attitudes can provide high-quality end-of-life health services and help dying patients achieve self-transcendence (8, 9). In addition, positive attitudes toward death will help health professional students alleviate their psychological distress caused by facing dying patients and death situations, which will indirectly improve their quality of palliative care (8, 9).

Over the past few decades, the importance of health professional students' death attitudes in the provision of high-quality care for dying patients has been increasingly recognized and emphasized, with extensive studies focused on death attitudes and associated factors. The bulk of previous studies has consistently demonstrated positive attitudes toward death among health professional students, with a higher score of neutral acceptance attitude reported in China (10, 11), Brasilia (12), Iran (13), and Turkey (14). However, there is also a small portion of studies, reporting negative attitudes toward death among health professional students, with lower scores on neutral acceptance attitudes (15). The divergencies in death attitudes may be explained by various factors that affect death attitudes in various studies. A recent literature review showed that most nurses and nurse students displayed positive attitudes toward death, and their attitudes were influenced by multiple factors, including age, gender, education level, knowledge of palliative care, death education, religious belief, and experiences related to death and palliative care (16). Specifically, many studies have cited death-related experiences as important factors affecting health professional students' attitudes toward death (16).

This study aimed to investigate the death attitudes of health professional students and analyze their associations with demographic characteristics and death-related experiences. Our results would provide a reference for the development of educational and interventional programs to improve health professional students' death attitudes in China, which, in turn, would enhance the quality of palliative care for dying patients in the future.

## Methods

### Study design and sampling

This was a multicenter, quantitative, cross-sectional study. A convenience sampling method was used to recruit health professional students from 14 medical colleges and universities to participate in the online questionnaire survey from March 2022 to May 2022. Eligible participants were health professional students of all grades (e.g., college, undergraduate, postgraduate, and doctoral students) who provided informed consent. The exclusion criteria were as follows: suspending schooling due to illness or other reasons, being diagnosed with mental disorders, not being Chinese. Finally, 1,044 eligible students with valid questionnaires were included in the study. The study was approved by the Ethics Committee of the Xiangya Hospital, Central South University (Approval No. 202207150).

### Data collection

A preliminary questionnaire was developed by our research team through a literature review, which was revised based on a panel discussion of five experts and a pilot study of 20 students. The final formal questionnaire was uploaded to “Sojump” (www.sojump.com). Sojump is one of China's biggest professional online platforms for questionnaire design, questionnaire distribution, data collection, and data analysis (17). An invitation email with a quick response (QR) code link to the Sojump questionnaire was forwarded to the participants by their teachers. The questionnaire took approximately 10 minutes to complete and was set to be submitted only after all the survey contents have been completed to ensure non-missing values.

### Demographic characteristics and death-related experiences

Demographic characteristics included gender, age, medical specialty, religious belief, education, place of residence, and whether only child or not. Death-related experiences had previous formal training in palliative care or death education, cardiopulmonary resuscitation (CPR), assisted breathing, and catheter feeding. Besides, participants were asked whether they had seen relatives or other people die, attended funeral/memorial services, or heard of ACP education.

### Death attitudes

Death attitudes were measured using the Chinese version of the DAP-R—originally developed by Wong et al. (3) to measure various aspects of death attitudes. In 2014, the DAP-R was translated into Chinese by Tang et al. (18) and showed good reliability and validity among Chinese nurses (19). The DAP-R includes 32 items under five subscales that represent five different attitudes toward dying and death (3): fear of death (seven items), death avoidance (five items), neutral acceptance (five items), approach acceptance (10 items), and escape acceptance (five items). Fear of death and death avoidance is attributed to negative death attitudes. Neutral acceptance, escape acceptance, and approach acceptance are attributed to positive death attitudes. Each item is rated on a 5-point Likert scale from 1 “strongly disagree” to 5 “strongly agree.” The subscale score is calculated by dividing the total subscale scores by the number of each subscale item. Each subscale score ranges from 1 to 5, with a higher score indicating a higher tendency to the subscale attitude toward death. In this study, the DAP-R survey showed good internal consistency with a Cronbach's α of 0.90.

### Statistics analysis

Statistics analysis was performed using SPSS 26.0 (IBM Corporation Armonk, New York, USA). Categorical variables were presented by numbers and percentages, while continuous variables were presented by means ± standard deviations (SD). Independent two-sample *t*-test was used to compare the means between two groups, while analysis of variance (ANOVA) was used to compare the means for more than two groups. A multiple linear regression model was used to analyze the influencing factors of death attitudes; *p* < 0.05 was considered statistically significant in the bilateral test.

## Results

### Death attitudes of health professional students

The scores of the total DAP-R scale, negative death attitudes, and positive death attitudes were 3.04 ± 0.48, 2.99 ± 0.69, and 3.08 ± 0.52, respectively (Table 1). The average scores for the five dimensions of the DAP-R, ranked from highest to lowest, were as follows: 3.94 ± 0.56 for natural acceptance, 3.02 ± 0.78 for death avoidance, 2.96 ± 0.73 for fear of death, 2.81 ± 0.65 for approach acceptance, and 2.74 ± 0.84 for escape acceptance.

**Table 1:** Descriptive data on death attitudes (*N* = 1,044).

**Variable**	**Min**	**Max**	**Mean ±S.D (total)**	**Mean ±S.D (item)**
Fear of death	7	35	20.75 ± 5.13	2.96 ± 0.73
Death avoidance	5	25	15.08 ± 3.89	3.02 ± 0.78
Neutral acceptance	5	25	19.68 ± 2.79	3.94 ± 0.56
Approach acceptance	10	50	28.12 ± 6.49	2.81 ± 0.65
Escape acceptance	5	25	13.71 ± 4.21	2.74 ± 0.84
Negative death attitudes	12	60	35.83 ± 8.30	2.99 ± 0.69
Positive death attitudes	20	100	61.51 ± 10.37	3.08 ± 0.52
Total score of death attitudes	32	160	97.34 ± 15.40	3.04 ± 0.48

### Univariate analysis of factors related to the death attitudes

Table 2 shows the results of the univariate analysis of factors related to positive death attitudes and negative death attitudes, respectively. The scores of negative death attitudes showed statistically significant differences by the following factors: age (*p* < 0.05) and religious belief (*p* < 0.05). The scores of positive death attitudes showed statistically significant differences by the following factors: age, medical specialty, previous training in palliative care (or death education), catheter feeding training, attending funeral/memorial services, and hearing of ACP; *p* < 0.05 for all these factors) (Table 2).

**Table 2:** Univariate analysis of death attitudes (*N* = 1,044).

**Variable**	***N* (%)**	**Negative death attitudes**	***t*/*F* value**	**Positive death attitudes**	***t*/*F* value**
**Gender**			0.563		0.059
Male	199 (19.1)	36.13 ± 9.54		61.55 ± 12.27	
Female	845 (80.9)	35.76 ± 7.98		61.50 ± 9.88	
**Age**			2.996^*^		2.106^*^
≤ 22 years	887 (85.0)	36.15 ± 8.11		61.79 ± 10.36	
>22 years	157 (15.0)	34.01 ± 9.07		59.90 ± 10.34	
**Medical specialty**			1.748		3.553^*^
Clinical medicine	103 (9.9)	34.40 ± 9.15		59.18 ± 10.75	
Nursing	913 (87.5)	36.00 ± 8.14		61.83 ± 10.25	
Others	28 (2.7)	35.50 ± 10.02		59.54 ± 12.00	
**Religion belief**			2.101^*^		0.298
Yes	56 (5.4)	38.09 ± 10.76		61.91 ± 13.81	
No	988 (94.6)	35.70 ± 8.12		61.49 ± 10.15	
**Education**			0.840		1.267
College student	20 (1.9)	35.45 ± 11.03		63.10 ± 15.17	
Undergraduate student	937 (89.9)	35.95 ± 8.10		61.65 ± 10.24	
MD student	61 (5.8)	34.23 ± 10.34		59.33 ± 11.10	
PhD student	26 (2.5)	35.58 ± 8.59		60.15 ± 8.80	
**Place of residence**			0.148		0.180
Rural	582 (55.7)	35.79 ± 8.11		61.49 ± 10.26	
Town	248 (23.8)	36.06 ± 8.23		61.80 ± 10.88	
Urban	214 (20.5)	35.66 ± 8.90		61.22 ± 10.09	
**Only child**			−0.551		0.968
Yes	265 (25.4)	35.58 ± 9.13		62.04 ± 11.43	
No	779 (74.6)	35.91 ± 8.00		61.33 ± 9.99	
**Previous training in palliative care (or**			−1.897		2.008^*^
**death education)**					
Yes	218 (20.9)	34.88 ± 9.34		62.76 ± 10.96	
No	826 (79.1)	36.08 ± 7.99		61.18 ± 10.19	
**CPR training**			0.838		0.334
Yes	593 (56.8)	36.02 ± 8.68		61.60 ± 10.59	
No	451 (43.2)	35.58 ± 7.76		61.39 ± 10.09	
**Assisted breathing training**			0.893		0.814
Yes	532 (51.0)	36.05 ± 8.68		61.77 ± 10.73	
No	512 (49.0)	35.59 ± 7.89		61.24 ± 9.99	
**Catheter feeding training**			−0.062		2.173^*^
Yes	555 (53.2)	35.81 ± 8.57		62.16 ± 10.63	
No	489 (46.8)	35.84 ± 7.98		60.77 ± 10.03	
**Have seen others die**			−0.901		0.505
Yes	721 (69.1)	35.67 ± 8.18		61.62 ± 10.12	
No	323 (30.9)	36.17 ± 8.55		61.27 ± 10.93	
**Have seen relatives die**			0.434		1.567
Yes	686 (65.7)	35.91 ± 8.23		61.87 ± 10.33	
No	358 (34.3)	35.67 ± 8.43		60.81 ± 10.43	
**Attending funeral/memorial services**			−0.024		2.807^*^
Yes	952 (91.2)	35.83 ± 8.14		61.79 ± 10.12	
No	92 (8.8)	35.85 ± 9.78		58.62 ± 12.38	
**Heard of ACP**			1.442		3.564^**^
Yes	399 (38.2)	36.30 ± 8.62		62.95 ± 11.48	
No	645 (61.8)	35.54 ± 8.08		60.61 ± 9.523	

Students with major in auxiliary clinical subjects, such as laboratory medicine, anesthesiology, and imaging.

MD, master's degree; PhD, doctoral degree; CPR, cardiopulmonary resuscitation; ACP, advance care plan; ^*^p < 0.05, ^**^p < 0.001.

### Multivariate analysis of factors associated with death attitudes

Multiple linear regression analysis was performed to explore independent influencing factors of negative death attitudes and positive death attitudes, respectively. Statistically significant variables in the previous univariate analysis were included in the multivariate analysis. The results identified two independent influencing factors of negative death attitudes: age (β = −0.31, *p* < 0.001) and religious belief (β = 2.76, *p* = 0.015), as well as three independent influencing factors of positive death attitudes: age (β = −0.42, *p* < 0.001), hearing of ACP (β = 2.21, *p* = 0.001), and attending funeral/memorial services (β = 2.69, *p* = 0.016) (Table 3).

**Table 3:** Influencing factors of death attitudes by multivariate analysis (*N* = 1,044).

**Variable**	**β**	**SE**	**Beta**	***P*-value**	**Exp (β) 95%Cl**
**Negative death attitude**					
Constant	39.31	1.857		< 0.001	35.668–42.954
Age	−0.31	0.072	−0.132	< 0.001	−0.452 to −0.168
Religious belief	2.76	1.132	0.075	0.015	0.538–4.979
*F* = 11.389 *P* < 0.001 *R*^2^ = 0.021					
**Positive death attitude**					
Constant	58.13	4.299		< 0.001	50.692–67.564
Age	−0.42	0.105	−0.144	< 0.001	−0.629 to −0.218
Nursing	−0.09	1.127	0.003	0.938	−2.299 to 2.124
Previous training in palliative care/death education	1.36	0.820	0.053	0.098	−0.253 to 2.965
Hearing of ACP	2.21	0.656	0.104	0.001	0.924–3.500
Catheter feeding training	0.94	0.682	0.045	0.167	−0.394 to 2.282
Attending funeral/memorial services	2.69	1.117	0.073	0.016	0.495–4.877
*F* = 7.815 *P* < 0.001 *R*^2^ = 0.043					

ACP, advance care plan; SE, standard error; CI, confidence interval.

## Discussion

Attitude toward death is a vital factor that can directly affect health staff's healthcare behaviors and quality of care (12). Thus, health professional students, the future health staff, should be trained to react and deal with dying patients positively and appropriately. Before the development of intern training programs, emphasis should be placed on understanding health professional students' attitudes toward death. Our study showed that the health professional students scored highest in the neutral acceptance dimension, indicating that most students tended to accept death as an integral part of life. This finding was consistent with most previous studies showing similar results in other parts of China (10, 11) and in other countries (12–14). One explanation may be that health professional students have received education in dialectical materialism and atheism in Chinese Universities. Moreover, some of them have already been exposed to patients' death during their clinical internship and thus are prepared for death experiences. It is, therefore, reasonable to see health professional students showing a high score of neutral acceptance attitude toward the phenomenon of death (20–22).

Death avoidance and death fear ranked in the middle of death attitudes' five dimensions, indicating that when facing death situations, it was common for some students to have negative emotions such as death escape and death anxiety. These students may lack a systematical and comprehensive understanding of death, as well as the experience of facing death. Thus, their fear of death might be evoked when confronting death for the first time (23). The least-scored dimensions of death attitudes were approach acceptance and escape acceptance. Approach acceptance is usually related to the belief that there is a better future after death, while escape acceptance is associated with the belief that death can end pain (23). The relatively low scores in these two dimensions may be related to culture and religion among Chinese health professional students.

To better deal with death through appropriate emotions and behaviors in the future career, it is of great importance to find out the influencing factors associated with health professional students' attitudes toward death. Our study further revealed different influencing factors associated with negative death attitudes and positive death attitudes. Negative death attitudes include death fear and death avoidance. The regression model showed that negative death attitudes of health professional students were influenced by age and religious beliefs. In this study, age was negatively related to both negative and positive death attitudes, with younger students ( ≤ 22 years of age) being more prone to have both negative and positive death attitudes. This finding was in line with previous studies showing that younger students had higher scores in multiple dimensions of death attitudes, such as death fear, death avoidance, and approach acceptance than older students (12, 24). Younger students were in the lower grade and had fewer opportunities to experience patients' death and were thus more likely to have positive and negative attitudes (24, 25). On the contrary, older students (>22 years of age) had higher education, such as master's and doctoral degrees, had more knowledge of clinical medicine, and had more experiences of patients' death; therefore, their attitudes toward death were more neutral.

Moreover, religious belief in the study was positively related to negative death attitudes. This was contrary to the previous findings (26), showing that religious beliefs were associated with diminished feelings of negative attitudes. However, another review showed a weak correlation between religious belief and death anxiety (27). Indeed, religion is a complex framework composed of multiple dimensions and conceptual components. Identified by Wulff in 1991 (28), religious belief consisted of four dimensions, including literal inclusion, literal exclusion, symbolic inclusion, and symbolic exclusion. The literal vs. symbolic dimension means that people interpret religion literally or symbolically, while inclusion vs. exclusion means whether they believe or they do not believe. Dezutter et al. (29) further explored that both literal inclusion and literal exclusion were positively correlated with negative death attitudes, indicating that people with a literal approach to religion had more difficulties facing death irrespective of whether they adopted a religious view or not. The reasonable explanation is that people with a literal approach to religion express less openness, more close mindedness, and less tolerance of ambiguity, leading to a defensive attitude toward death (29). Therefore, there is a hypothesized intermediary role of openness, mindfulness, and tolerance of ambiguity in the relationship between literal thinking and death attitudes. In our study, we did not analyze the multidimensional framework of religious belief since there were only a few (5.4%) religious students. Based on the aforementioned theory, these religious students may be less open, more close minded, and less tolerant of ambiguity, more contributing to their negative death attitudes. In future research, religious belief should be analyzed under a multidimensional framework with a larger sample of religious students to further validate such a hypothesis.

Conversely, positive death attitudes were shown to be positively correlated to hearing of ACP and attending funeral/memorial services, a finding consistent with previous studies (8–11, 16). ACP is defined as the process that allows individuals, at the status of consciousness and being capable of decision-making, to specify in advance how they want to be treated when they enter the dying state or the progressive deterioration of their health condition is out of control (30). In brief, ACP is a critical, person-centered, comprehensive, and continual process increasing patients' satisfaction and quality of life. It was also reported that ACP could relieve nursing staff's distress by enhancing their knowledge and competence in end-of-life healthcare (26). Therefore, ACP empowers health professionals to cope with dying patients and alleviate their death fear (31). However, the current education and practice of ACP in China are at the initial stage, and a standard ACP nursing training system has not been established (32). Our result sheds light on the relationship between ACP and positive death attitudes that even hearing about ACP could enhance the positive attitudes toward death. This finding highlights the need to design a complete ACP education to promote positive death attitudes among health professional students during their clinical study and practice.

Finally, our study revealed that health professional students who had attended funeral/memorial services were prone to have positive death attitudes. This was in line with a previous study showing that health professional students who attended funeral/memorial services were more likely to have a neutral attitude toward death. At the same time, those without such experiences tended to hold more negative death attitudes (33). Research further showed that continuous exposure to death via attending funeral/memorial services prompted individuals to accept the inevitability of their own mortality (34). Therefore, attending a funeral ritual may be recommended as part of death education to promote positive death attitudes among health professional students (32). Combined with our previous discussion on ACP education, we strongly recommend that attending funeral/memorial services should be incorporated into ACP education in China.

## Limitations

There are some limitations in this study. First, the sample contained mostly female undergraduate students with major in nursing. In contrast, other groups, i.e., male medical students with master's and above degrees, were much less presented in the sample, which might affect the study generalization. Second, all the data were self-reported, i.e., the death-contact experiences reported by health professional students, which may be subject to recall bias. Third, the cross-sectional study design may preclude any causal inferences between death attitudes and associated factors. Additionally, religious belief was not stratified in this study due to the small number of religious students.

## Conclusion and implications

This is the first large-scale multicenter study to investigate death attitudes and associated factors among health professional students in China. Health professional students in the study generally tend to accept death neutrally. Moreover, their attitudes toward death are closely related to age, religious belief, hearing of ACP education, and attending funeral/memorial services. Our results reinforce the need to include death and palliative care education in healthcare courses as early as possible to foster the knowledge, attitudes, and behaviors of palliative care among health professional students, especially the younger students. The incorporation of multiple psycho–pedagogical approaches, such as ACP education in health professional students' routine curriculum or training programs, along with experiences with death-related activities such as attending funeral/memorial services, will enhance their positive attitudes toward caring for dying patients. These measures will help health professional students better prepare for death situations and thus improve their quality of palliative care in their future careers. In addition, further research is needed to examine the association between religious beliefs and death attitudes using a multidimensional framework with a larger religious sample. This study provides a multifaceted approach to promote the death attitudes of health professional students and a timely strategy to enhance ACP education for health professional students in China.

## Data availability statement

The original contributions presented in the study are included in the article/supplementary material, further inquiries can be directed to the corresponding author.

## Ethics statement

The studies involving human participants were reviewed and approved by the Ethics Committee of the Xiangya Hospital, Central South University (Approval No.202207150). Written informed consent to participate in this study was provided by the participants' legal guardian/next of kin. Written informed consent was obtained from the individual(s) for the publication of any potentially identifiable images or data included in this article.

## Author contributions

HH: formal analysis and project administration. HH and FZ: supervision, writing—original draft, and modification. All authors: conceptualization, study design, coordination of data collection, acquisition in data, and funding acquisition. All authors contributed to the article and approved the submitted version.
